# Concerning BPC-157, a natural pentadecapeptide, that acts as a cytoprotectant and is believed to protect the gastro-intestinal tract (GIT)

**DOI:** 10.1007/s10787-025-01882-z

**Published:** 2025-08-04

**Authors:** Michael Whitehouse

**Affiliations:** 1 School of Environmental Sciences, Griffith University, Brisbane South, QLD Australia; 2 School of Medicine, Griffith University, Southport, QLD Australia; 3 P.O. Box 6168, Woolloongabba, QLD 4102 Australia

**Keywords:** Proline-rich peptides, Organ/ cellular protection, Pharmacogenetics, Toxicology

## Abstract

This article discusses the lengthy review by Pedrag Sikiric and twenty one (21) co-authors in Inflammopharmacology (2024) 32:3119–3161.

## Preamble

This commentary is one reader’s response to a remarkable review in this Journal by Predrag Sikiric and 21 colleagues in Zagreb, Croatia, detailing the pharmacological potential of this apparently (almost) ubiquitous pleiotropic polypeptide BPC-157 (Sikiric et al. 2024).

The abbreviation, ‘BPC’, refers to the peptide’s designation as a body protection compound. The 2024 review contains over 130 references specifically referring to this body peptide published over a period of thirty years. BPC-157 was first described as a peptide in gastric juice with beneficial effects published in *Journal of Physiology*, Paris (Sikiric et al. 1993).

The peptide was subsequently described as a cytoprotectant with the radical property of being stable in gastric juice and therefore accessible for both gastric and intestinal protection after oral administration.

This is both provocative and amazing!

Here is a natural product, probably of animal origin, readily available by ex vivo chemical synthesis and identified as a hormone-like gastro-protectant (think prostacyclin) and stimulant of healing/wound repair (again think prostaglandins particularly resolvins) and with no reported toxicity!

It almost seems too good to be true!

Am I mistaken in presenting such an assessment?

## A synopsis of some queries about BPC-157

- If BPC-157 is such an outstanding natural therapeutic agent (perhaps an endogenous hormone), why are there so few reports of its potential bio-regulant significance based on human studies? BPC-157 has long been available as a synthetic chemical (DIAGEN d.o.o, Ljubljana, Slovenia) with the amino acid composition GEPPPGKPADDAGLV, which presumably was used for many of the experiments presented in this review in the form of two tables: Table 1 (9 plus pages) summarising beneficial effects in the context of GIT protection; and Table 2 (14 plus pages) describing beneficial effects via cellular protection extended to other organ therapy.
- A cogent question is, ‘how might it be marketed’ with so little likelihood of being patentable, being a natural product?
- Are there any reports of bioactivities of modified pentapeptides e.g. acetylation of the N-terminal glycine (perhaps using ketene) or of the C-terminal valine by esterification or preparation of amides, which might be useful pro-drugs with some promise of patent protection?
- Are all 15 amino acid residues required for ‘cytoprotective activity’, assuming there is a quick, reliable in vitro assay available? This is following the conventional strategy of obtaining a useful structure–activity relationship (SAR) so beloved by medicinal chemists.
- Using such assays, what is the actual potency of BPC-157 that might be compared with that of other cytoprotectants (of lower molecular size)?
- Is it feasible to use this peptide clinically in combination therapies, e.g. with known gastro-erosive medications, such as prednisone and other oral steroids?
- Do all the alternative cytoprotectants (discussed in the introduction to this review) interact with the same, or very similar, cellular receptors? Can they synergise with BPC-157?
- How significant is the presence of four (4) proline residues (GEPPPGKPADDAGLV) within this one molecule? Does the triple proline sequence determine the polypeptide’s stability in vivo, i.e. resisting inactivation by trypsin, chymotrypsin and various endo-cellular, catheptic proteases?
- Gingerpain, a protease present in edible ginger (*Zingiber officiale*), can hydrolyse prolyl-containing peptide sequences (C. Hawkens, pers. comm). Does gingerpain destroy BPC-157? (This is a ‘knotty challenge’ as proline residues introduce ‘kinks’ into peptide sequences.)
- Are there specific transporters which ensure export of the polypeptide from the cells of origin? OR
- Is BPC-157 merely an indigestible protein-fragment / sequence (pepsin resistant) remaining after gastric, extracellular digestion?
- Do any libraries of protein structures indicate the presence of the 3P sequence in biopolymers contained in foods consumed by humans or domestic animals; perhaps ‘highlighted’ as having extra nutritional value (being a source of natural gastro-protectants)?
- Are there studies of BPC-157 labelled with ^3^H or ^14^C to indicate biodistribution (after various alternate routes of administration), catabolism in vivo, excretion etc.? These would allow some ‘(gu)estimate’ of a useful half-life. Can these be modified by other dietary components and do they vary, not only because of dietary factors but also by genetic endowment? Pharmacogenetics could be very significant in this context. This is all quite important in considering large-scale clinical trials, particularly as a preventive medication to treat gastric and/or intestinal disorders.
- It is implied in the review that this pentapeptide is both organ-protective and also promotes remarkable healing after injury. Are the receptors and fundamental anabolic mechanisms the same in both these situations, i.e. normal (physiological) pre-injury health and the pathological post-injury context prior to healing?

## The bottom line

This polypeptide certainly needs to be re-evaluated to determine if it is hormonal like some other proline-rich peptides, e.g. the casomorphins found in milk (especially the BCM-7) (Woodford 2010, 2021) and systemin from plants.

The composition of BCM-7 shows three prolyl residues, i.e. YPFPGPI (Woodford 2010) and is stable enough to be found in human urine. BCM-7 is a septa-peptide that is catabolised by the enzyme dipeptidyl-peptidase IV in normal individuals, but which may be absent or defective in autistic people and possibly in people suffering from schizophrenia and also diabetes (J. Hill cited in Woodford 2010 as Appendix 2). These are certainly provocative conclusions. A very similar opioid peptide, gliadorphin-7, derived from the partial digestion of gliadin, a component of gluten, has the molecular structure YPQPQPF, also with 3 prolyl residues (Woodford 2021).

Systemin is an 18 amino polypeptide released after injury to the leaves of plants, which activates plant defensive system(s) (Ryan and Pearce 1998) after attack by insects or pathogens. The structure of systemin, as recorded in the Merck Index, is AVQSKPPSKRDPPKMQTD-H^+^, with diprolyl residues at positions 6 & 7 and 12 & 13.

Perhaps we now need to reconsider the ambivalent pathophysiological properties of proline-containing peptide / proteins regarding their metabolism in health and disease. This is a growing area of Pathobiodynamics, and Conditional Toxicity, so important in determining health, longevity and the future effective use of medications.

## Data Availability

There is no original data or data source used other than the published reference materials referred to in this piece.
